# Editorial: Cancer stem cells as attractive targets for breast cancer therapy

**DOI:** 10.3389/fonc.2023.1151742

**Published:** 2023-03-03

**Authors:** Sherif Abdelaziz Ibrahim, George W. Yip, Martin Götte

**Affiliations:** ^1^ Department of Zoology, Faculty of Science, Cairo University, Giza, Egypt; ^2^ Department of Anatomy, Yong Loo Lin School of Medicine, National University of Singapore, Singapore, Singapore; ^3^ Department of Gynecology and Obstetrics, Münster University Hospital, Münster, Germany

**Keywords:** cancer stem cells, cancer initiating cells, breast cancer, metastasis, epithelial-mesenchymal transition, tumor microenvironment, immunotherapy

Breast cancer is the most prevalent cancer that affects females worldwide. It accounts for approximately 31% of cancers in women (1). Breast cancer can be classified into various intrinsic subtypes based on the expression of estrogen receptor (ER), progesterone receptor (PR), human epidermal growth factor receptor 2 (HER2), and the proliferative index Ki-67: luminal A (ER-positive, PR-positive, HER2-negative, Ki-67 with less than 20% positivity); luminal B-HER2- negative (ER-positive, HER2- negative, and either PR-low or Ki-67 with more than 20% positivity); luminal B HER2-positive (ER-positive or PR-positive, HER2-positive or amplified, and regardless of Ki-67 status); HER2-enriched (ER-negative, PR-negative, HER-2-positive or amplified, and regardless of Ki-67 status); and triple-negative (ER-negative, PR-negative, and HER2-negative) breast cancer (2–4). Despite recent advances in breast carcinoma treatment, including chemotherapy, radiotherapy, and hormone (endocrine) therapy, as well as innovations in targeted therapeutics and immunotherapy (5), development of treatment resistance and tumor recurrence are still major challenges. In contrast to differentiated cells, cancer stem cells (CSCs) or tumor initiating cells (TICs), which constitute a subpopulation of cells within the tumor bulk, have been linked to tumor relapse and resistance to conventional therapy (6–9). Therefore, targeting CSCs has emerged as a promising therapeutic strategy against cancer, including breast cancer. CSCs or TICs possess unique features, including self-renewal, expression of multidrug resistance proteins, high proliferative capacity, efficient DNA repair capability, resistance to apoptosis, and overexpression of therapeutic efflux proteins (10–15). Breast CSCs are characterized by expression of cell surface makers CD44^+^/CD24^-^ and/or increased detoxifying aldehyde dehydrogenase (ALDH) activity (6, 16). Breast CSCs are influenced not only by cancer-cell autonomous factors but also by diverse cues derived from the tumor microenvironment (TME) (17).

This Research Topic collection comprises four papers (two original research, one perspective, and one review article) coauthored by 28 researchers. We aimed to provide original research and opinion pieces on breast CSCs, focusing on new molecular markers and therapeutic targets, crosstalk between tumor microenvironmental cells and breast CSCs, and signaling pathways involved in breast CSC regulation.

Discovering new molecular markers expressed by breast CSCs is of particular interest for breast cancer management. Yang et al. showed that two splicing variants of very-low-density lipoprotein receptor (VLDLR-I, II), a member of the low-density lipoprotein receptor (LDLR) superfamily involved in lipid metabolism, are overexpressed in breast CSCs relative to non-breast CSCs. Using gain-and loss-function experiments, the authors further verified the biological role of VLDR-I and -II expression in regulating breast CSC functions *in vitro* and *in vivo*, namely xenograft tumor growth, tumor cell proliferation, and spheroid and colony formation. Suppression of breast tumor growth is mediated by VLDR knockdown-induced cellular quiescence in a ligand-independent manner. Increased expression of VLDR is correlated with elevated energy production and ribosome biogenesis, as well as associated with poor clinical outcome in breast cancer patients.

Breast CSCs express many immunosuppressive molecules (e.g., the immune checkpoint ligands-programmed death-ligand (PD-L)1 and PD-L2), which impede the functions of natural killer and T- immune cells and result in promoting immune evasion and stemness. Ruiu et al. addressed in their perspective article the immunological properties of breast CSCs and questioned the suitability of their exploitation as targets for immunotherapeutics (e.g., vaccines, adoptive cell therapy and chimeric antigen receptor (CAR)-T cells, monoclonal antibodies, and small molecules) based on preclinical studies and clinical trials. The authors reported encouraging results of some clinical trials. However, the applicability of immunotherapies to target breast CSCs is still under study and has not been proven yet.

Inflammatory breast cancer (IBC), the deadliest and highly metastatic form of breast cancer, is enriched with CSCs in comparison with non-inflammatory types of breast cancer. Tumor-associated macrophages (TAMs), an important component of IBC TME, play an essential role in IBC pathogenesis and regulate breast CSCs. Macrophages infected with the human cytomegalovirus (HCMV) act as “mobile vectors” for virus dissemination into breast tissues. Mohamed et al. demonstrated that the secretome (containing IL-6, IL-8, and MCP-1) of TAMs infected with HCMV induced IBC SUM149 cells to undergo proliferation, invasion, colony formation and breast CSC-related gene expression *via* activation of STAT3, AMPKα, PRAS40, and SAPK/JNK compared to untreated SUM149 cells.

It is well known that breast CSCs are regulated by development-associated signaling pathways, including Wnt, Notch, and Hedgehog pathways. Walker et al. discussed and provided evidence for the role of prostaglandin E2 pathway in breast CSCs using published transcriptome data. The authors also addressed the relevance of targeting the prostaglandin E2 pathway for breast cancer treatment, especially for CSCs-enriched tumor subtypes.

Overall, the publications within this Research Topic collection provide further support for the breast CSC concept and provide novel avenues of targeting a key tumor cell population associated with therapeutic resistance and recurrence.

## Author contributions

All authors made substantial contribution to the work and approved it for publication.
